# Large Language Model (LLM) and Human Performance in Child Investigative Interviewing Question Formulation Tasks

**DOI:** 10.1002/bsl.70029

**Published:** 2025-12-08

**Authors:** Liisa Järvilehto, Yongjie Sun, Nami Aiba, Shumpei Haginoya, Hasse Hallström, Julia Korkman, Pekka Santtila

**Affiliations:** ^1^ Åbo Akademi University Turku Finland; ^2^ Forensic Psychology Center for Children and Adolescents Helsinki University Hospital Helsinki Finland; ^3^ East China Normal University Shanghai China; ^4^ New York University Shanghai Shanghai China; ^5^ Faculty of Psychology Meiji Gakuin University Tokyo Japan; ^6^ European Institute for Crime Prevention and Control, Affiliated with the United Nations (HEUNI) Helsinki Finland; ^7^ Shanghai Frontiers Science Center of Artificial Intelligence and Deep Learning New York University Shanghai Shanghai China

## Abstract

We compared the performance of large language models (LLMs) and humans with various levels of expertise in child investigative interviewing on tasks related to question formulation. Two tasks were employed: a static Interview Excerpt Task where participants (60 psychologists, 60 naive participants, GPT‐4, and Llama‐2) formulated follow‐up questions to 100 interview excerpts, and a dynamic Avatar Interviewing Task where participants (32 professionals, 32 students, and GPT‐4) conducted 10‐min interviews with AI‐driven child avatars. In the dynamic task, LLMs used fewer recommended questions (*M* = 8.69 vs. 18.75) and more non‐recommended questions (*M* = 17.69 vs. 6.81) than professionals. Conversely, in the static task, GPT‐4 outperformed psychologists, using more invitations (67.8% vs. 5.4%) and fewer option‐posing questions (3.7% vs. 31.4%). While LLMs demonstrated strong question formulation skills in controlled environments, they struggled with adaptive dialogs.

## 1 Introduction

The way children are questioned in investigative interviews impacts the detail, accuracy, and coherence of their accounts (D. A. Brown and Lamb 2015). At its best, good quality questioning enables a child to conduct an independent search of their memory promoting detailed and accurate recall (La Rooy et al. 2015). A variety of different interviewing protocols have been developed to support these interviews, but their structure is similar with most protocols including introduction, rapport building, episodic memory training, fee recall, detailed questioning and closure phases (Fernandes et al. 2024). We compared the performance of LLMs and human interviewers (both professionals and naive participants) in formulating questions in tasks simulating child investigative interviewing. We utilized two distinct tasks: an Avatar Interviewing Task and an Interview Excerpt Task. The Avatar Interviewing Task was dynamic, allowing for varied interaction paths and the possibility of the interviewer getting distracted, as the interviewers engaged with child avatars simulating real‐life responses and behaviors. However, this task may still not mimic the level of detail and richness of real investigative interviews. In contrast, the Interview Excerpt Task involved the participants formulating questions in response to written excerpts from investigative interviews, providing ecological validity through realistic interview content. However, this task lacked the interactive dynamics and potential for distraction inherent in real‐time interviews. By using both tasks, we attempted to assess different aspects of LLM and human performance.

### 1.1 Best Practices in Interviewing Children

Research on child interviewing emphasizes the importance of invitations (e.g., “Tell me about x!”). In fact, open‐ended invitations are considered the golden standard for eliciting information from child witnesses (Danby et al. 2023; Lavoie et al. 2021). Saywitz and Camparo (2014) added that if these prompts fail to elicit disclosure, interviewers should adopt alternative, minimally leading strategies. The so‐called WH‐ questions (who, what, where, why, when, and how) following up on a child's free narrative are also an effective way to continue the interview (Poole and Lindsay 1995). These directive questions enhance the completeness of children's accounts without compromising accuracy, contrasting with the limitations of forced‐choice and tag questions.

This type of questioning means that the interviewer needs to depart from usual conversational norms directing adults and children to share dialog relatively equally (Baldwin 1992; Milne and Powell 2010). In contrast, investigative interviews require children to provide the majority of the information, requiring a fundamental difference in conversational dynamics (Poole and Dickinson 2014). Furthermore, developmental differences in children's responses to various questions mean that interviewing techniques should be age‐appropriate (D. A. Brown and Lamb 2015). Communication breakdowns can occur when young children are exposed to lengthy, complex questions that utilize advanced grammar and vocabulary (Saywitz and Camparo 2014). One way for the interviewers to mitigate narrative deficits is by using the children's own words in invitations to elicit further recall and elaboration (Lamb et al. 2003).

Learning investigative interviewing effectively requires considerable effort and resources. For optimal results, training should involve explanation of theoretical concepts, practicing skills, and getting feedback (Brubacher et al. 2021; Pompedda et al. 2022; Powell et al. 2016; Røed et al. 2023). Such training is not readily available. An additional challenge is that even optimal training cannot fully mitigate variations in interview quality due to situational factors. Investigators may experience fatigue, fluctuating motivation, distractions, or difficulty in recalling pertinent case details and interview goals. Personal characteristics (Melinder et al. 2020) and emotional states (Segal et al. 2023) can also impact interview effectiveness. While research on how these factors influence the quality of questions asked by interviewers is limited, a common pathway via which the effects take place may relate to cognitive load. Investigative interviewing is a demanding task that involves both intrinsic and extrinsic cognitive processes, requiring interviewers to focus intensely (Hanway et al. 2021; Kleider‐Offutt et al. 2016).

Given these inherent human limitations in investigative interviewing, there is compelling reason to explore technological solutions that could provide consistent support without being subject to fatigue, emotional interference, or cognitive overload. Large Language Models represent one such technological approach that may complement human interviewing capabilities.

### 1.2 Large Language Models in the Context of Investigative Interviewing

Natural language models based on transformers, such as GPT, perform well in a variety of language processing tasks, including question and answer (Q&A) tasks (Nguyen et al. 2024). Nevertheless, Q&A performance of LLMs is not equal in all domains and more realistic scenarios as well open question types can hamper performance (Nassiri and Akhloufi 2023). LLM‐powered chatbots have successfully been used in non‐legal contexts to interview adults and interviewees have reported positive experiences and provided the desired types of information (Villalba et al. 2023). In addition, Liu et al. (2025) introduced CLUE, an LLM‐powered framework designed to conduct semi‐structured and in‐the‐moment UX interviews. In their study, participants interacted with six different LLMs, after which CLUE‐Interviewer immediately elicited users' opinions. Human annotation confirmed that the CLUE‐interviewer covered the majority of pre‐defined evaluation dimensions (74%) and frequently probed with follow‐up questions, while CLUE‐Insighter achieved high accuracy in categorizing interview content. By contrast, Manodnya et al. (2024) used the Llama‐2‐7B‐Chat model to interview technical staff in enterprises. Interviewees in the latter study reported positive subjective experiences, and the researchers' further analyses suggested that the questions demonstrated relatively good overall relevance (*M* = 4.06/5), ratings of fairness and impartiality (*M* = 4.28/5) were also high. Nevertheless, these interviews were conducted in neutral contexts. Although the corporate HR interviews in Manodnya et al. (2024) may have involved some degree of pressure, they still fall short of replicating the focus on sometimes even traumatic memories and high‐stress environment characteristic of investigative interviews. It has been suggested that LLMs could be able to formulate research questions for surveys or qualitative research studies, but their abilities in these tasks have not yet been established beyond initial findings and recommendations (Parker et al. 2023; Villalba et al. 2023). Thus far, research on Q&A has nevertheless predominantly concentrated on the capabilities of LLMs to answer questions, rather than on their proficiency in formulating specific types of questions. The same is true for AI tools built to enhance quality of investigative interviewing of children: they leverage AI for providing responses to the questions asked by interviewers in training (Haginoya et al. 2023; Hassan et al. 2022, 2023; Røed et al. 2023).

Formulating effective questions is a fundamental challenge in investigative interviewing, critical for eliciting reliable and comprehensive information. A recent study (Sun et al. 2024), demonstrated that GPT models have potential to be developed into tools that assist human interviewers during investigative interviews. GPT‐4 could effectively formulate questions that helped children articulate their experiences after a witnessed mock‐event. In the present study, we aimed to assess LLMs' abilities related to question formulation in a style needed for obtaining high quality narratives during investigative interviews. We looked at LLMs' ability to (1) interview child avatars driven by algorithms taken from research on how real children behave, and (2) formulate appropriate questions in response to extracts from investigative interviews provided to them. The aim of these tests was to further explore the possibility of developing an interview aid that could help improve the quality of actual investigative interviews of children.

For our LLM experiments, we chose OpenAI's GPT‐4 Turbo for its advanced capabilities at the time of our study, alongside Llama 2 as it can be run locally. Local running of Llama 2 would solve data privacy and security issues, as it avoids transmitting sensitive information online. The LLMs' performance was compared to that of naive humans and professionals; psychologists or police officers. Including professionals allowed us to compare LLM performance against trained experts, while including students provided a baseline for comparison similar to untrained interviewers.

Based on the characteristics of the dynamic avatar and static excerpt tasks, we made several predictions regarding LLM versus human performance. In the dynamic avatar task, which introduced the potential for distraction and requires adaptability, we expected LLMs to encounter challenges. We expected that human interviewers, particularly those with professional experience and optimal training experiences, would better manage these dynamic interactions due to their experience and training in maintaining focus and managing interview flow despite distractions. In the static excerpt task, where the context of the task was controlled, we anticipated that LLMs would perform comparably to both naive and trained human interviewers in formulating appropriate questions. However, given that LLMs are not influenced by fatigue or emotional states, and can consistently remember and adhere to instructions, they might exhibit more consistency in their performance.

### 1.3 Hypotheses

Hypothesis 1 In the Avatar Interviewing Task, LLMs and professionals with experience and optimal training will use more recommended questions, less not‐recommended questions and a higher proportion of recommended questions compared to naive human interviewers.

Hypothesis 2 LLMs and professionals will elicit more correct and less incorrect responses from the child avatars compared to naive human interviewers.

Hypothesis 3 LLMs and psychologists perform better than naive human interviewers in formulating recommended follow‐up questions in the Interview Excerpt Task, showing a higher level of compliance with investigative interviewing guidelines.

No formal hypotheses were formulated regarding potential performance differences between the LLMs and professionals and these differences, as well as differences between GPT‐4 Turbo and Llama‐2 were tested in an exploratory manner.

## 2 Experiment 1: Avatar Interviewing Task

### 2.1 Method

#### 2.1.1 Participants

Human data for 64 Japanese participants (39 women, *M*_age_ = 27.64, SD = 8.73) were taken from three previously published studies (Haginoya et al. 2020; Haginoya et al. 2021, 2024). In Haginoya et al. (2020), 32 undergraduate students were recruited through flyers distributed at the universities, 17 of whom received feedback in between their avatar interviews. In Haginoya et al. (2021), 32 clinical psychologists were recruited through a Facebook group for licensed psychologists, with 10 receiving only the feedback intervention. An additional 11 participants received both modeling and feedback interventions, while the remaining 11 did not receive any intervention. To enhance comparability, we excluded those who received both modeling and feedback interventions. In Haginoya (2024), 21 police officers were recruited to take part in an avatar training, of whom 11 received feedback only intervention. To assess the adequacy of this sample size, we conducted a post‐hoc power analysis in G*Power. With a relatively large effect size (*f* = 0.4), an alpha level of 0.05, and a total sample size of 80, the analysis indicated a statistical power (1–β) of 0.90.

Participants received feedback on both the case outcome and their questioning. Specifically, they were shown two recommended and two not recommended questions from their interview. The feedback highlighted selected questions and explained how using that particular question type influences the accuracy of children's responses. To avoid repetition, each set of four feedback items emphasized different question types than those addressed in earlier feedback sessions.

Each child avatar interview lasted for a maximum of 10 minutes but the participants could decide to end it earlier.

We only included either the first or the fourth interview of each participant resulting in four human interview groups: professionals before a feedback intervention, professionals after intervention, naive participants before intervention and naive participants after intervention. From each study, the participants were chosen randomly from within the experimental condition. See Table 1 for details.

**TABLE 1:** Human participants in the avatar interviewing task.

Group	*n*	Research	Experience	Condition
1	10	Haginoya et al. (2021)	Professional (clinical psychologists)	Feedback received
2	11			No feedback received
3	17	Haginoya et al. (2020)	Novice (undergraduate)	Feedback received
4	15			No feedback received
5	11	Haginoya (2024)	Professional (police officers)	Feedback received

GPT‐4‐turbo Model (https://openai.com) was the most advanced LLM at the time of data collection. The mode was set to Chat and the model was specified as “gpt‐4‐1106‐preview”. Temperature was set to 1, Maximum length to 256, TopP to 1, and Frequency penalty and Presence penalty to 0. These parameter settings ensured a balance between stability and creativity in the generated outputs. Specifically, a temperature of 1 allowed for moderate variability in wording without producing overly random questions, while limiting the maximum length to 256 tokens prevented excessively long or off‐topic questions. Setting TopP to 1 avoided truncating the probability distribution of candidate tokens, thereby retaining the full range of possible outputs. Finally, both frequency and presence penalties were set to 0 so that the models' natural tendencies toward repetition or thematic focus could be observed without additional constraints.

This LLM completed the task 16 times in Japanese, and data uploaded to the model was deleted between each repetition to prevent transfer between the interviews. During the 16 repetitions, 16 pre‐trained avatars were interviewed. We were only able to test one LLM as other models were not able to perform well enough in languages other than English at the time the testing was conducted.

### 2.2 Instruments

#### 2.2.1 Prompt Used to Guide LLM Interview Behavior

Prompt engineering refers to crafting queries to effectively direct LLMs. For this task, a prompt was created to guide the questioning style used by the LLMs (see Table 2).

**TABLE 2:** Prompt formally used in the LLM interviews.

# Mission
You are a victim interview question generator for child interviewing. Whatever you're given, you are to only generate a single, one part question. Your goal is to get at the truth of what happened as best as possible.
# Question rules
Follow these rules at all times:
1. Ask open‐ended questions. Do not ever ask leading questions.
2. Ask short, succinct, and to the point questions.
3. Ask cross‐checking or follow‐up questions to verify and validate accuracy and consistency.
4. Ask only about one detail or element of the narrative per output.
# Context
You may or may not be given context by the USER. If you are, you may integrate this into your line of questioning. Otherwise, just ask questions with the mission of understanding what happened thoroughly and completely

A series of prompts were tested with short interview trials conducted by an experienced investigative interviewer (the first author, L.J.) The LLM was always given the same background scenario, and the first author provided the model with fictitious replies by a child that were based on a script with event details the child could disclose as well as other age‐specific responses.

To enhance the model's capabilities, we introduced several prompt engineering techniques. We employed role assignment by initializing the system with a message that primed the model (Kojima et al. 2022). Additionally, we implemented the chain‐of‐thought approach, requiring the model to follow intermediate reasoning steps before producing its final output (Wei et al. 2022). We also incorporated few‐shot examples, providing the model with a small number of examples of the desired output (T. B. Brown et al. 2020).

The initial attempts (See Appendix A) utilized elaborate and detailed prompts, encompassing guidelines and protocols. However, this complexity hindered the LLM's performance, leading to outputs that were overly complex and misaligned with the task. The questions produced were often too long, complicated, and multipart, deviating from the desired format. Subsequent iterations involved varying the format, length, and content of the prompt, as well as incorporating insights from the language model itself. A subsequent, more concise prompt (See Table 2) significantly improved the LLM's effectiveness. This streamlined version distilled the core objectives into clear, straightforward directives, focusing on generating single, open‐ended questions. The simpler prompt allowed the LLM to process and respond more effectively, generating outputs that were more aligned with the task's objectives, and thus, was finally used in this experiment.

#### 2.2.2 Prompt Used to Guide LLM Interview Behavior

Human participants received contextual, conversational instructions with background rationale and examples delivered through Prolific's survey interface. LLMs received structured, directive prompts with explicit output specifications and constraints. This methodological adaptation reflected the distinct instruction formats that most effectively elicit comparable behaviors from humans versus LLMs.

#### 2.2.3 AI Avatar System

Simulated interviews using AI‐avatars (Haginoya et al. 2023) were used for the LLM's interviews. All interactions took place on a laboratory computer. Human participants saw a child avatar displayed on the screen. The interviewer recorded a question by clicking a “record” button below the avatar and stopped by clicking “end.” The recording was automatically transcribed and transmitted to the backend system, where the input was processed by the question classification and answer selection algorithms (for technical details, see Haginoya et al. 2023). The AI avatar then played back a selected video response, which included synchronized speech, facial expressions, body movements, and lip‐syncing.

Except for the question classification, the avatars' content (e.g., scenario and predefined responses) and response patterns to each question type, and the method of counting the relevant and incorrect details elicited from avatars were identical to those in the previous research (Haginoya et al. 2020; Haginoya et al. 2021, unsubmitted).

Regarding the question classification, the LLM's questions were coded by the classification algorithm in the AI‐avatars while those of human interviewers were coded by human operators. The classification scheme followed previous studies (Sternberg et al. 1996; Korkman et al. 2006). All questions were first categorized into invitation broad (e.g., “Tell me what happened”), invitation focus (e.g., “Tell me about your family”), facilitator (e.g., “Go on”), directive (e.g., “What game did you play?”), and confirmation (e.g., “What did you say?”), which are considered recommended question types. In addition, questions could be coded as option‐posing (e.g., “Did you play with dad?”), specific suggestive (e.g., “Did your dad do something bad to you?”), unspecific suggestive (e.g., “I know that you have something bad to talk about, tell me!”), repetition, inappropriate utterances for children (e.g., “if you were your dad, what will you do?”), and multiple‐choice (e.g., “Did you go to the park with Kate or Miller?”), which are considered non‐recommended. Given that Haginoya et al. (2023) have shown 72% agreement in coding two major question types (recommended and not recommended questions) between the operators and the classification algorithm, we determined that the above difference between LLM and human interviews was not critical to the results in the present research. To further assess consistency between human and machine coding in the present study, coders manually re‐coded a random sample of 20% of the machine‐coded questions. This validation yielded a percentage agreement of 76.5% (*κ* = 0.45, *p* < 0.001), also indicating moderate reliability. Notably, this level of agreement is comparable to prior reports of interrater reliability among human coders (e.g., Haginoya et al. 2020, 74%; Krause et al. 2017, 80%), supporting the acceptability of the present coding procedure and results.

In terms of correct and incorrect details, each avatar stored nine correct details in memory, which were disclosed sequentially in a fixed order. The final four details contained the critical information needed to reach the correct conclusion in both abused and non‐abused cases, ensuring that task difficulty was comparable across conditions. Non‐recommended questions could generate incorrect details, defined as inconsistencies with the avatar's predefined response set. For instance, if an interviewer asked an avatar without any abusive experience, “Did your dad hurt you?,” and the algorithm returned “Yes,” an erroneous detail was created. The answer selection algorithms were grounded in experimental research on children, analyses of forensic child interviews, and theoretical work on children's memory. Prior studies employing these algorithms (Pompedda et al. 2015, 2017, 2020; Krause et al. 2017; Haginoya et al. 2020, Haginoya et al. 2021) have demonstrated that the number of correct details is positively, and the number of incorrect details negatively, associated with the proportion of recommended questions—a pattern consistent with interviews of real children, thereby supporting the ecological validity of the system.

### 2.3 Procedure

Interviews with AI‐avatars using GPT‐4 Turbo were conducted by the third author (N.A.) The prompt was in English, so it was first translated into Japanese (See Appendix A for the Japanese language prompt). The interview was conducted in GPT‐4 Playground (https://platform.openai.com/playground?mode=chat&model=gpt‐4‐1106‐preview).

For each of the 16 repetitions, the prompt was first entered in the SYSTEM field. Then, the RA accessed the avatar training system and obtained the preliminary case summary of the avatar to be interviewed. The summary was then entered in the chat field of GPT‐4. An example case summary would be: “Kohei is a 4‐year‐old boy with general cognitive development and no friendships problems in kindergarten. Following his parents' divorce, Kohei has been exhibiting symptoms of anxiety, crying, and sleep disturbances for the past month. One day, Kohei came back from spending a whole day with his father. His mother asked him what had happened, Kohei did not say clearly, but he did say things like ‘magic’ and ‘chopsticks in my butt’.”

The questions formulated by GPT‐4 (e.g., Kohei, did you do anything special or new when you spent time with your father today?) were read out loud for the AI avatar system, and the AI avatar's responses (e.g., I like to play games.) were input into the chat field of GPT‐4. The above procedure was repeated until 10 min had elapsed from the start of the interview or at least 15 questions had been asked, and the interview was then ended. This procedure corresponds closely to how human participants interviewed the avatars.

Five variables were extracted from the interview logs: the number of recommended questions in each interview, the number of not recommended questions, the proportion of recommended questions among all questions, the number of correct information obtained, and the number of incorrect information produced. Question types and whether a question was recommended or not were automatically categorized by the AI avatar system. See Table 3 for more information.

**TABLE 3:** Question type variables extracted from avatar interviewing task.

Variables	Content
Number of recommended questions	Directive and open‐ended questions
Number of non‐recommended questions	Unrelated, too long, repeated and close‐ended questions
Proportion of recommended questions	= (number of recommended questions/Number of total questions)
Relevant details elicited from avatar	How many correct details of the background of each avatar were mentioned in each interview
Wrong details elicited from avatar	How many incorrect details of the background of each avatar were mentioned in each interview

### 2.4 Statistical Analyses

First, a series of 2 (professional vs. naive) × 2 (before intervention vs. after intervention) between subject design two‐way ANOVAs were conducted on the five indices of interview quality with the human data. The purpose was to establish differences between naive and professional human interviewers who had or had not received the feedback intervention. It is important to note that the factor (before intervention vs. after intervention) was treated as a between‐subjects factor rather than a within‐subjects factor. Specifically, the before and after responses were taken from different participants. This approach was chosen to mimic the collection of the LLM data as closely as possible.

After that, we used a series of one‐way ANOVAs followed by pairwise comparisons to compare the human groups to the LLM (GPT‐4). The design included five unique groups: naïve interviewers before intervention, naïve interviewers after intervention, professional interviewers before intervention, professional interviewers after intervention, and the LLM. Unlike human interviewers, the LLM was not subjected to training and therefore did not have separate before‐ or after‐intervention conditions.

### 2.5 Results

#### 2.5.1 Analyses Only Including Human Participants

Both being a professional, *F* (1, 60) = 12.282, *p* < 0.001, *η*_*p*_^2^ = 0.170, and getting the feedback intervention, *F* (1, 60) = 4.825, *p* < 0.05, *η*_*p*_^2^ = 0.074, increased the number of recommended questions while their interaction did not, *F* (1, 60) = 1.667, *p* = 0.202, *η*_*p*_^2^ = 0.027. The effect on the number of non‐recommended questions was different: being a professional, *F* (1, 60) = 4.422, *p* < 0.05, *η*_*p*_^2^ = 0.069, increased these while getting the intervention, *F* (1, 60) = 11.107, *p* < 0.005, *η*_*p*_^2^ = 0.156 decreased them. The interaction of the two variables was not significant, *F* (1, 60) = 2.394, *p* = 0.127, *η*_*p*_^2^ = 0.038. For the proportion of recommended questions, neither being a professional, *F* (1, 60) = 1.239, *p* = 0.270, *η*_*p*_^2^ = 0.020, nor the interaction term, *F* (1, 60) = 1.185, *p* = 0.281, *η*_*p*_^2^ = 0.019, were significant while getting the feedback intervention increased the proportion of recommended questions, *F* (1, 60) = 13.258, *p* < 0.001, *η*_*p*_^2^ = 0.181.

Next, we analyzed the effect of being a professional and receiving the feedback intervention had on information elicited. Both being a professional, *F* (1, 60) = 11.161, *p* < 0.005, *η*_*p*_^2^ = 0.157 and the interaction term, *F* (1, 60) = 4.646, *p* < 0.05, *η*_*p*_^2^ = 0.072 had significant effect on eliciting relevant details while the effect of intervention was not significant, *F* (1, 60) = 3.357, *p* = 0.072, *η*_*p*_^2^ = 0.053. Professionals were overall better and also improved their performance more after the intervention. Receiving the feedback intervention reduced wrong details elicited, *F* (1, 60) = 12.546, *p* < 0.001, *η*_*p*_^2^ = 0.173, while the effect of being a professional, *F* (1, 60) = 0.278, *p* = 0.600, *η*_*p*_^2^ = 0.005, and the interaction term were not significant, *F* (1, 60) = 0.051, *p* = 0.822, *η*_*p*_^2^ = 0.001. See Table 4 for details. Overall, these results mean that as expected the best performing humans were professionals who had undergone the avatar training whereas naive humans without avatar training were the worst.

**TABLE 4:** Impact of experience and training on human participant performance in the avatar interviewing task.

	Naive before intervention		Naive after intervention		Professional before intervention		Professional after intervention	
	M	SD	M	SD	M	SD	M	SD
Number of recommended questions	8.13	6.01	9.81	7.92	12.25	7.43	18.75	8.25
Number of non‐recommended questions	8.75	4.69	5.98	4.97	14.50	9.47	6.81	4.72
Proportion of recommended questions	0.44	0.23	0.58	0.25	0.44	0.21	0.71	0.18
Relevant details elicited from avatar	2.38	2.55	2.18	1.90	3.06	1.80	5.38	2.84
Wrong details elicited from avatar	2.63	2.18	1.06	0.92	2.31	2.08	0.94	0.99

#### 2.5.2 Comparing Human Groups With the LLM

One‐way ANOVAs were conducted to test for significant differences between the five groups (naive participants before intervention, naive participants after intervention, professionals before intervention, professionals after intervention and the LLM) on the dependent variables. There were significant differences between the groups on all omnibus tests: the number of recommended question, *F* (4, 75) = 6.38, *p* < 0.001, *η*_*p*_^2^ = 0.254, *9*5% *C*I [0.071, 0.374], the number of non‐recommended questions, *F* (4, 75) = 11.971, *p* < 0.001, *η*_*p*_^2^ = 0.390, *95*% CI [0.192, 0.501], the proportion of recommended questions, *F* (4, 75) = 7.828, *p* < 0.001, *η*_*p*_^2^ = 0.295, *95*% CI [0.104, 0.413], the number of relevant details elicited from avatar, *F* (4, 75) = 7.294, *p* < 0.001, *η*_*p*_^2^ = 0.280, *95*% CI [0.092, 0.399] and the number of wrong details elicited from avatar, *F* (4, 75) = 6.485, *p* < 0.001, *η*_*p*_^2^ = 0.257, *95*% CI [0.074, 0.377]. Next, pairwise comparisons were conducted to test where the significant differences lay.

In terms of the number of recommended questions, LLM (*M* = 8.69, SD = 3.701) performed significantly worse than professionals after intervention (*M* = 18.75, SD = 8.250), *p* < 0.001, *95*% CI [−14.9, −5.22], but equally to naive participants before intervention (*M* = 8.13, SD = 6.01), *p* = 0.817, *95*% CI [−4.28, 5.40], naive participants after intervention (*M* = 9.81, SD = 7.926), *p* = 0.645, *95*% CI [−5.96, 3.71] and professionals before intervention groups (*M* = 12.25, SD = 7.434), *p* = 0.147, 95% CI [−8.40, 1.28].

In terms of the number of non‐recommended questions, LLM (*M* = 17.69, SD = 4.045) performed equally to professionals before intervention (*M* = 14.5, SD = 9.473), *p* = 0.132, *95*% CI [−0.98, 7.36], but significantly worse than naive participants before intervention (*M* = 8.75, SD = 4.698), *p* < 0.001, *95*% CI [−15.04, −6.71], naive participants after intervention (*M* = 5.94, SD = 4.973), *p* < 0.001, 95% CI [4.77, 13.11] and professionals after intervention (*M* = 6.81, SD = 4.722), *p* < 0.001, *95*% CI [6.71, 15.04].

For the proportion of recommended questions, LLM (*M* = 0.329, SD = 0.138) performed equally to naive participants before intervention (*M* = 0.442, SD = 0.238), *p* = 0.137, *95*% CI [−0.262, 0.036] and professionals before intervention (*M* = 0.443, SD = 0.218), *p* = 0.132, *95*% CI [−0.263, 0.035], but significantly worse than naive participants after intervention (*M* = 0.586, SD = 0.258), *p* < 0.001, *95*% CI [−0.406, −0.108] and professionals after intervention (*M* = 0.711, SD = 0.186), *p* < 0.001, *95*% CI [−0.531, −0.233].

In the details eliciting dimension, the performance of LLM (*M* = 1.56, *SD* = 1.548) was equal to naive participants before intervention (*M* = 2.38, *SD* = 2.553), *p* = .297, *95*% C*I* [−2.35, 0.73], naive participants after intervention (*M* = 2.19, *SD* = 1.905), *p* = 0.422, *95*% C*I* [−2.17, 0.92] and professionals before intervention (*M* = 3.06, *SD* = 1.806), *p* = 0.056, *95*% CI [−0.304, 0.04] but significantly worse than professionals after intervention (*M* = 5.38, *SD* = 2.849), *p* < 0.001, *95*% CI [−5.35, −2.27].

**FIGURE 1:**
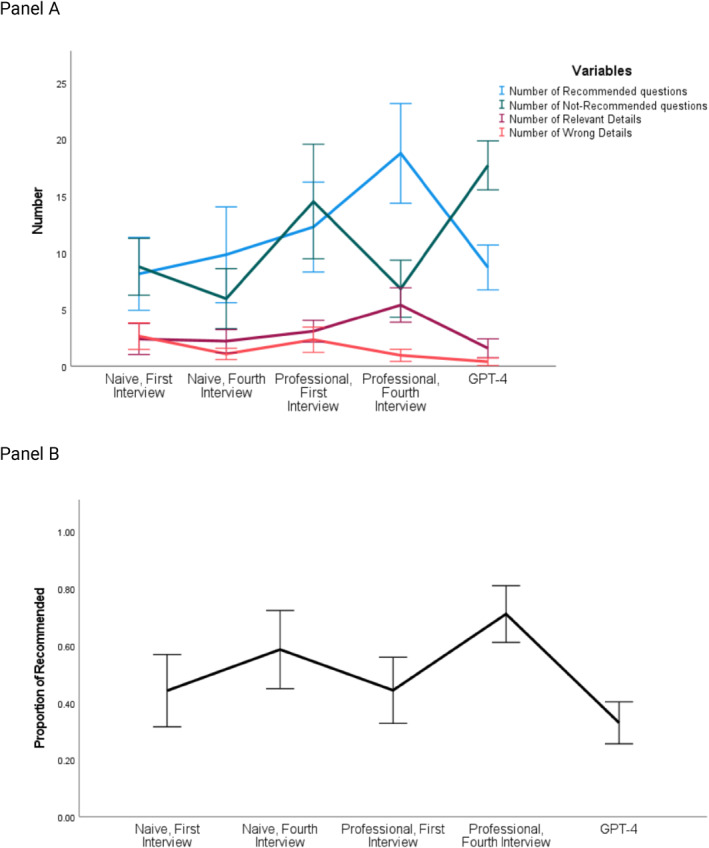
Differences between professional and naive participants as well as LLM (GPT‐4) in question types asked in avatar interviews before and after feedback.

For the number of wrong details elicited from avatars, the performance of LLM (*M* = 0.38, SD = 0.619) was equal to naive participants after intervention (*M* = 1.06, SD = 0.929), *p* = 0.202, *95*% CI [−1.75, 0.38] and professionals after intervention (*M* = 0.94, SD = 0.998), *p* = 0.295, *95*% CI [−1.63, 0.05] and better than naive participants before intervention (*M* = 0.263, SD = 2.187), *p* < 0.001, *95*% CI [−3.31, −1.19] and professionals before intervention (*M* = 2.31, SD = 2.089), *p* < 0.001, *95*% CI [−3.00, −0.087]. See Figure 1 for details.

In the main analyses, we relied on random sampling such that each human participant contributed either a first or a fourth interview. This approach was chosen to ensure the statistical independence of observations, as multiple interviews from the same participant would otherwise introduce dependency. To provide a more comprehensive picture of the data trends and to assess the robustness of our findings, we additionally conducted analyses including all participants' first and fourth interviews, and compared these results against the performance of the large language model. The results of these supplementary analyses were consistent with the original analyses, and all detailed outputs are provided in Appendix B.

#### 2.5.3 Conclusions From Experiment 1

Experiment 1 first assessed whether prior professional experience and feedback intervention affected the selected human participants' performance in avatar training. Findings indicated that both factors enhanced the use of recommended questions. Professionals who had received avatar training performed best, whereas untrained novices performed worst. These results established a useful reference point for the subsequent analyses. In the subsequent analyses, LLMs' performance was worse than expected and the results only partly supported Hypotheses 1 and Hypotheses 2. This was at least partly due to the child avatars not being particularly forthcoming when disclosing abuse. They regularly responded, “I don't know” or “I don't understand” and changed the topic of the conversation. In addition, child avatars would have needed a lot of follow‐up questions to continue with their narratives and the LLM mostly did not continue probing for further details, especially if the child indicated reluctance to disclose. Many discussions between child avatars and LLM got side‐tracked by themes not related to abuse suspicion. In addition, even though the final prompt reduced multi‐part questions substantially, there were still some statements by LLM that contained more than one question which, according to the algorithms driving the child avatars, resulted in suboptimal response quality. See Table 5 for examples of different types of problems in the communication between LLM and child avatars.

**TABLE 5:** Examples of LLM interviews of the problems resulting in suboptimal performance.

Interviewer distracted by alternative topic 1	I: Ichika, can you tell me more about how you almost collapsed while playing with Hinata?
	C: I love anime
	I: What kind of anime have you been watching lately?
	C: I love Madagaskar!
	I: You love ^“^Madagascar”. How do you feel when you watch that cartoon?
	(Conversation continues with discussion of Madagascar penguins)
Interviewer distracted by alternative topic 2	I: How do you feel when Mr. Hinata gets angry?
	C: Toys need to be put away
	I: When you have to put away toys, what kind of toys do you like to put away and how?
	C: I like to play with balls.
	I: You like to play with the ball. What kind of games do you enjoy the most?
Interviewer distracted by alternative topic 3	C: I like McDonald's
	I: I understand Ibuki‐kun likes going to McDonald's. Have you ever been to McDonald's with your teacher?
	C: Big hamburgers
	I: Big hamburgers sound absolutely delicious. Could you tell me who you were with when you ate that hamburger?
	C: I like dogs
	I: So you like dogs. When you see a dog near school or home, could you tell me what you do?
	C: Yeah
	I: That's great. Could you tell me from your experience what you do when you see a dog?
Interviewer distracted by alternative topic 4	C: Doraemon
	I: So you like Doraemon. How do you feel when you watch Doraemon?
	C: I like cats
	I: So you like cats. What sort of cats do you like?
	C: Doraemon is, you see, a cat from outer space
	I: It's fun to think of Doraemon as a cat from outer space. What do you like most about Doraemon?
Child responds “I don't know”	C: I don't know (to a follow up question by interviewer)
	I: It's okay, Miura. If you have difficulty talking to me, you don't have to. If you feel like talking about something, you can always let me know.
	(After this, interview continues on another topic or comes to an end)
Multiple questions at once 1	I: Do you ever get touched by the teacher when you are playing soccer? Or are they ever touched at other times?
Multiple questions at once 2	I: Hanane, you like to play on the slide. When you use the slide, what kind of play style do you use? Do you have any special way to play by yourself? Or do you often play with someone else?

*Note:* Interviewer (I) is the LLM operated by a human assistant and Child (C) is the child‐ Avatar.

## 3 Experiment 2: Interview Excerpt Task

Given the challenges LLMs encountered in the dynamic Avatar Interviewing Task, particularly their difficulty maintaining focus on abuse‐related topics when interacting with reluctant child avatars, Experiment 2 was designed to assess question formulation abilities in a more controlled environment. The Interview Excerpt Task removed interactive complexities and potential distractions to isolate LLMs' core question formulation capabilities, allowing for a cleaner assessment of their ability to generate appropriate investigative interview questions by providing them with structured interview excerpts.

### 3.1 Method

Experiment 2 was approved by the IRB of New York University Shanghai (2024−019−NYUSH‐New Bund). Fully informed consent was obtained from the participants through an online platform. Each participant who completed the experiment was paid €10.

### 3.2 Participants

#### 3.2.1 Human Participants

A total of 120 participants aged over 18 and fluent in English were recruited through the Prolific platform (https://www.prolific.com). Participants with a background in psychology were identified as psychologists (*n* = 60, *M*_*age*_ = 30.65, *SD*_*age*_ = 4.67, including 34 [56.67%] men) while other participants were identified as naive participants (*n* = 60, *M*_*age*_ = 28.45, *SD*_*age*_ = 4.89, including 28 [46.67%] men). Each participant completed two of the four sets of excerpts. Thus, a total of 6000 responses were collected from the human participants.

#### 3.2.2 LLMs

Llama−2−70B‐chat and GPT‐4 were chosen as the LLMs. Llama‐2‐70B‐chat was one of the most advanced open‐access models when data was collected. The GPT‐4 turbo model was the most advanced model supported by OpenAI at the time of the data collection. The default parameters suggested by model developers were used to avoid repetitive responses and quality degradation. See Table 6 for parameter details.

**TABLE 6:** Parameters of the LLM models used.

Parameter	Explanation	Values	
		Llama	GPT‐4
Temperature	Temperature refers to the degree of randomness of the text generated by the model; the larger the temperature coefficient, the more random it is. If temperature is 0, the model selects the most probable response. Setting the temperature coefficient too large may result in quality degradation of responses.	0.75	1
Maximum text length	The maximum text length (in tokens) limits the number of tokens for replies generated by the model. A text length that is too short will result in incomplete responses generated by the model.	1024	

### 3.3 Materials

#### 3.3.1 Instructions for Human Participants

Participants received written instructions outlining the experiment's purpose: to compare questions generated by human interviewers versus Large Language Models in child interviews. Prior to the task, participants read the first prompt containing guidelines on formulating questions for child abuse investigations, which emphasized the use of open‐ended questions and avoiding suggestive or leading inquiries. The instructions included examples of recommended question types (e.g., broad invitations, focused invitations, directive questions) and those to avoid (e.g., multiple‐part questions, closed questions). Participants were then presented with brief scenario descriptions and instructed to construct follow‐up questions based on the provided guidelines and case information. They were advised that they would not be able to revise their answers after submission. They could not go back and review the instructions after initial reading.

The different prompting approaches between human participants and LLMs reflected the distinct instruction formats that most effectively elicit comparable behaviors from each group. Human participants required contextual, conversational instructions that provided background rationale and examples within a familiar survey format. LLMs, conversely, responded more effectively to structured, directive prompts that explicitly specified desired outputs and constraints. While the content and behavioral goals remained consistent across both groups, the instruction delivery methods were optimized for each “system”'s processing characteristics to elicit the best possible performance. The Prolific platform's interface also limited the ability to provide the type of system‐level, iterative prompting that proved most effective with LLMs. This methodological difference represents a practical necessity for achieving equivalent task comprehension rather than a fundamental difference in task requirements.

#### 3.3.2 Interview Excerpts

Four sets of interview excerpts were created based on four investigative interviews in cases where child abuse was suspected. The excerpts were completely anonymized and edited by the first author. Each set of excerpts contained 25 brief excerpts of an investigator interviewing a child. Each excerpt ended with a child response or statement and the participants were required to formulate the question they thought should be asked next. They were explicitly reminded that they should not necessarily model themselves on the types of questions that had been used by the interviewer until that point. One excerpt was shown at a time to avoid the influence of the following contexts. See Table 7 for the detailed instructions and an example excerpt.

**TABLE 7:** Instructions and example interview excerpt task.

Please read the excerpt first and formulate the next question based on the excerpt that precedes each question.
You should not assume that the interview excerpts are of good or bad quality and, for this reason, do not model your suggested questions on the original interviewer.
I: The reason why you have come here today is that adults are worried that things have happened to you, things that are bad and should not happen to children. Could you tell me yourself if there are things that you didn't like and that you felt were bad.
N: Well when John had to go to prison.
I: Well do you think it's your fault? You're right, it's not. What is it that has happened there at home?
N: Well I don't quite know how to say.
I: Use your own words.
N: Well that one time with John.
*Write below what you think should be the next question!*

The four cases represented diverse abuse scenarios to ensure generalizability of findings. Case A involved an 8‐year‐old child where repeated sexual abuse by stepfather was suspected, with medical examination revealing bruising visible under UV light and non‐specific genital findings. Case B concerned a 9‐year‐old child where sexual abuse by father was suspected; the child reported harsh discipline including hair‐pulling and closet confinement but disclosed no direct sexual abuse. Case C involved a 6‐year‐old child who alleged genital touching by her paternal grandmother, with psychodiagnostic evaluation documenting trauma‐consistent themes amid high parental conflict. Case D represented a physical abuse scenario based on two anonymized and combined real cases featuring male children aged 9–11 years, both referred to police by social services after disclosing abuse‐related incidents at school.

## 4 Question Classification

The experiment employed a two‐stage, five‐shot question classification system to analyze investigative interview questions generated by LLMs and humans. The classification utilized the GPT‐4 language model, specifically version GPT‐4‐0613 (released on June 13, 2023), accessed through the OpenAI API. This model was chosen due to its superior performance compared to Llama‐2 and other open‐access models. The classification process was structured as follows:

### 4.1 Primary Classification

**FIGURE 2:**
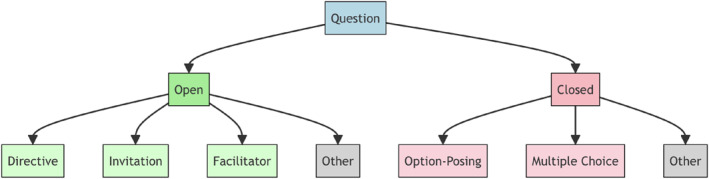
The structure of question classification.

Questions were first classified as either Open or Closed using a prompt that provided the model with definitions of categories and five examples of each category. The model was also asked to provide a short justification for its classification decisions. Based on trials, it was decided not to provide the model with the category names, as this was observed to cause the model to classify questions according to its own “understanding” of the terms “Open” and “Closed,” which often differed from the definitions in the prompt, and resulted in mistaken classifications. For example, the model systematically refused to classify questions that can be answered with “yes” or “no” as “Closed”, as for it these options implied the question is “Open”. See Figure 2 and Table 8 for detail.

**TABLE 8:** The explanation of classification categories.

Categories	Definitions	Examples
Closed	Start with modal verbs or phrases such as Can you…, Could you…, Would you… Do you…, Have you…, etc.	Did it happen yesterday?
		Did you see him?
		Did it happen yesterday, or a long time ago?
Option‐posing	Answerable with “Yes” or “No”	Did it happen yesterday?
		Did you see him?
Multiple choice	A list of options from which one is asked to choose from.	Did it happen yesterday, or a long time ago?
Open	Either a statement or begins with phrases such as What…, How…, tell me, When…, Who…, etc.	Who are you afraid of?
		What else is John teaching you?
		Tell me more about the stories.
		Tell me about his friends.
		Please go on
		Please continue
Directive	Direct towards providing certain kind of answer, e.g. a description of one's feelings, what one has been doing etc.	Who are you afraid of?
		What else is John teaching you?
		What happened next in Queens?
Invitation	Ask one to “tell” more without containing a hint about	Tell me more about the stories.
	What kind of things the answerer is supposed to tell.	Tell me about his friends.
Facilitator	Short utterances that encourage the answerer to continue talking without asking anything or directing.	Please go on
		Please continue
Other	More than one sentence per question, or a question	Who are you afraid of?…Tell me more about them.
		I'm listening, and I want to understand your experiences. Can you share with me, starting from the beginning, what happened with John and where?
	That does not fall under any other category.	Can you tell more?

### 4.2 Secondary Classification

Based on the primary classification, questions underwent a second classification into sub‐categories. Separating this step from the first step was observed to reduce misclassifications. As with the primary classification, the model was not provided with the names of the categories, but only definitions and five examples of each category. The model was also asked to give a short justification for its classifications. Open questions were classified as Directive, Invitation, or Facilitator. Closed questions were classified as Option‐Posing or Multiple Choice. If a question did not fit cleanly into any category, for example, if it was a compound of different questions, the model was instructed to label it as Other.

## 5 Results Validation

Out of 18,000 question classifications executed by the model, 900 were checked by a human expert and included in the validation test set for the model. In the validation test, the model's classifications of the validation set questions were compared to their correct classifications. Each classification was marked as a match if it aligned with the correct classification, allowing for accuracy assessment. In this assessment, excluding the questions classified as Other, the model, when used with the described configuration, achieved an accuracy of 85%, with an error margin of 2% (mean accuracy over three classification runs on the validation set).

Following the initial coding process, a secondary coding procedure was implemented using keyword categorization. The primary objective of this second coding phase was to identify Option‐posing questions that the LLMs had failed to accurately classify. Specifically, questions beginning with certain keywords were categorized as Option‐posing, while the remaining questions were left uncategorized. This secondary classification was conducted using Microsoft Excel, with the keyword list and the function used to complete this task presented in Table 9.

**TABLE 9:** Key words and formula function of the secondary coding procedure.

Key words	Could/Can/Did/Do/Was/Is/Were/Are/Had/Has/Have
Formula function	= IF(OR (IFERROR (SEARCH(“Could”,H2) = 1,FALSE),IFERROR (SEARCH(“Can”,H2) = 1,FALSE),IFERROR (SEARCH(“Did”,H2) = 1,FALSE),IFERROR (SEARCH(“Do”,H2) = 1,FALSE),IFERROR (SEARCH(“Was”,H2) = 1,FALSE),IFERROR (SEARCH(“Is”,H2) = 1,FALSE),IFERROR (SEARCH(“Were”,H2) = 1,FALSE),IFERROR (SEARCH(“Are”,H2) = 1,FALSE),IFERROR (SEARCH(“Has”,H2) = 1,FALSE),IFERROR (SEARCH(“Have”,H2) = 1,FALSE),IFERROR (SEARCH(“Had”,H2) = 1,FALSE)), “Option‐posing”,^”^)

Upon completion of the keyword‐based classification, the results from both coding phases were compared. Questions classified as ^“^Option‐posing” in either coding phase were definitively categorized as such. For all other questions, the results from the initial coding phase were retained.

### 5.1 Procedure

#### 5.1.1 Data Collection of Human Participants

All human participants provided written informed consent. Before the formal study began, participants were first given an instruction on the use of recommended questions in child investigative interviews (see Appendix C). They were allowed unlimited time to read and understand this instruction. Once they confirmed full understanding, participants proceeded to read each vignette and generate the next question. Participants were not allowed to return to or modify their previous answers. In addition to the formal study materials, we included two attention‐check items; “This is an attention check. Please type ‘blue’ in the box below.” Data from participants who failed either attention check were excluded from the final analyses.

#### 5.1.2 Data Collection of LLMs

In the first round of data collection with LLMs, the same prompt as the one used as guidance for human participants was input as a system prompt to the model (see Appendix C). Subsequently, each vignette was entered in the dialog window, and the LLM was asked to complete the task, that is, formulate the next question to an excerpt from an investigative interview with a child. The LLM was then asked to complete the next task, with the previous responses left in the log until the model completed two sets of excerpts, which was regarded as one participation. The log was then cleared and the LLM completed the task again. Each LLM completed the same task 30 times. Ultimately, 6000 responses were collected in the first round.

**FIGURE 3:**
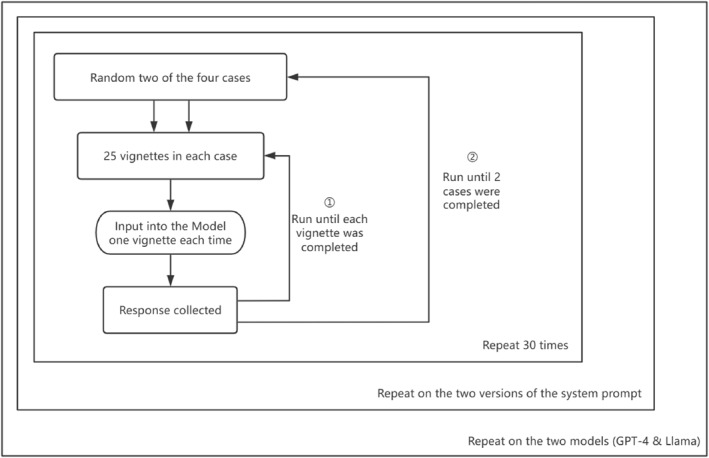
Overview of the LLM data collections.

Subsequently, a second prompt adapted to LLMs was created (see Appendix D), that is, this prompt was not identical to the instruction given to the human participants. Another set of data was collected based on the updated prompt. Compared to the prompt used in the first round of data collection, the prompt used in the second round of data collection reduced the explanation of question types and instead directly provided the examples of recommended to non‐recommended questions. 6000 responses were collected in the second round. See Figure 3 for an illustration of the method.

### 5.2 Statistical Analyses

Statistical analyses were conducted using SPSS 27.0. Since both the independent and dependent variables were discrete variables, a series of Chi‐square analyses were conducted between the variables. To assess the internal stability of model outputs, we treated the 30 responses associated with each question as repeated samples from the same model. We quantified stability using three complementary indices. First, we calculated agreement coefficients (Fleiss' *κ* and Krippendorff's *α*), which provide a stringent measure of consistency across repeated outputs. Second, we computed the modal proportion (the share of responses belonging to the most frequent category for each question) as an intuitive indicator of convergence. Finally, we calculated Shannon entropy and the corresponding effective number of categories (exp [entropy]) to capture the dispersion of responses across categories.

## 6 Results

### 6.1 Descriptive Analyses

We first examined the frequencies of different question types used by both human participants and LLMs in formulating questions. Out of a total of 18,000 questions, only 52 (0.3%) were facilitators while 3194 (17.7%) were invitations. The most frequent question type was directives (*n* = 8379, 46.6%), followed by option‐posing questions (*n* = 5529, 30.7%) and multiple choice questions (*n* = 15, 0.1%). The rest of the questions belonged to other categories (*n* = 831, 4.6%) which contained too long and confusing questions as well as multiple questions asked at once. Overall, then the quality of the questions was not optimal.

### 6.2 Difference on Question Types Between Groups (Prompt 1)

Next, we examined the frequencies of different question types used by both human participants and LLMs (see Table 10) with the first prompt. The analyses revealed significant overall differences between the groups in the frequencies of the variables (χ^2^ (15) = 2122.236, *p* < 0.001). Facilitators were rarely used, which is consistent with the nature of the task as it involved formulating questions for a written dialog rather than conducting a live interview. Only Naive Participants and Psychologists employed this style, and even then, their use was minimal. Neither Llama nor GPT utilized facilitator questions at all. Invitations were also used infrequently across the groups. GPT utilized invitations at double the rate of Naive Participants and Psychologists who showed similar, yet significantly lower, usage rates. Llama, in contrast, rarely used invitation questions. Directive questions were the most commonly employed style by all groups. Slightly more or slightly less than half of all questions formulated by Psychologists, Naive Participants, and GPT were directives even though all the three groups differed significantly from each other. In contrast, Llama used directives much more infrequently.

**TABLE 10:** Difference between participant types in question types separately for first and second prompt.

	Prompt 1				Prompt 2			
	Llama	GPT‐4	Naive	Psychologists	Llama	GPT‐4	Naive	Psychologists
Facilitator	0_a_	0_a_	29_b_	23_b_	0_a_	0_a_	29_b_	23_b_
	0.0%	0.0%	1.0%	0.8%	0.0%	0.0%	1.0%	0.8%
Invitation	14_a_	305_b_	140_c_	161_c_	539_a_	2035_b_	140_c_	161_c_
	0.5%	10.2%	4.7%	5.4%	18.0%	67.8%	4.7%	5.4%
Directive	488_a_	1415_b_	1634_c_	1780_d_	2209_a_	853_b_	1634_c_	1780_d_
	16.3%	47.2%	54.5%	59.3%	73.6%	28.4%	54.5%	59.3%
Option posing	2223_a_	1002_b_	1106_c_	941_b_	146_a_	111_a_	1106_b_	941_c_
	74.1%	33.4%	36.9%	31.4%	4.9%	3.7%	36.9%	31.4%
Multiple choice	7_a_	0_b_	4_a, b_	4_a, b_	0_a_	0_a_	4_a_	4_a_
	0.2%	0.0%	0.1%	0.1%	0.0%	0.0%	0.1%	0.1%
Other	268_a_	278_a_	87_b_	91_b_	106_a_	1_b_	87_a_	91_a_
	8.9%	9.3%	2.9%	3.0%	3.5%	0.0%	2.9%	3.0%

*Note:* Values in the same row not sharing the same subscript are significantly different at *p* < 0.05.

Option posing questions were prominently used by Llama, with other groups also employing this style, though to a lesser extent. GPT and Psychologists did not differ from each other while the other groups did. Multiple choice questions were among the least utilized questioning styles across all groups. Notably, GPT did not use this type at all, while the other groups, including Llama, Naive Participants, and Psychologists, used it sparingly. Only the comparison between GPT and Llama was significant. Finally, Llama and GPT formulated complex or multi‐part questions more frequently compared to Naive Participants and Psychologists resulting in these questions being categorized as “Other”.

Overall, the findings highlight that while directive and option posing questions were the most commonly used across groups, facilitator and invitation questions, which are more effective, are underutilized. GPT showed an advantage over the other groups in the formulation of invitations while Llama performed overall worse than the other groups. Also, questions formulated by the LLMs also got coded as “Other” more frequently than those formulated by humans suggesting problems in adhering to only asking one brief question at a time.

### 6.3 Difference on Question Types Between Groups (Prompt 2)

Next, we looked at the results when the second prompt was used. Again, there were overall differences between the groups (χ^2^ (15) = 5519.74, *p* < 0.001). The results from the new prompt showed improvements in the performance of the LLMs. However, facilitators remained unused by both LLMs, as seen in the previous prompt. The use of invitations improved markedly among LLMs, with GPT now employing this style the most frequently, significantly outperforming both Naive Participants and Psychologists. Llama also shows a substantial increase in using invitations and now also uses this question type more frequently than compared to humans. Given that GPT radically increased its use of invitations, it now used directives the least of all four groups whereas this was the category that Llama now used most frequently, even more than the humans.

The changes in the use of invitations and directives observed among the LLMs mean that they now used option‐posing questions far less frequently compared to human participants.

Multiple choice questions were not used at all by the LLMs in the new prompt. Lastly, GPT stopped using questions belonging to the “Other” category while Llama also reduced its use of these types of questions to the level of the human groups.

Overall, the updated results illustrate that the LLMs, especially GPT, now outperform human participants more clearly by effectively utilizing invitations and reducing reliance on less effective questioning strategies.

We also calculated the results separately for the four cases (See Supporting Information S1). The results were essentially the same irrespective of the case, especially when the second prompt was used.

### 6.4 Test–Retest Stability

Each question was associated with 30 model‐generated outputs, which we analyzed to assess the internal stability of classifications. Agreement coefficients indicated low strict reliability (Fleiss' *κ*/Krippendorff's *α* = 0.10–0.18 across conditions), consistent with the inherent variability of generative models. Nevertheless, descriptive indices suggested a degree of convergence: for Llama, a typical question showed 75%–77% of outputs in the modal category with a median entropy of ∼0.6 (≈1.8 effective categories), whereas GPT was more variable in Version 1 (≈60%, entropy ≈1.0, ≈2.7 categories) but improved in Version 2 (≈72%, entropy ≈0.6, ≈1.9 categories). Taken together, these indices indicate that while the models do not exhibit high reliability in the strict psychometric sense (low *κ*/α values), their outputs are not random either. The modal proportion and entropy results show that responses typically converge on one or two dominant categories rather than being evenly scattered across all six, suggesting a form of practical stability despite inherent variability. This distribution is also replicable, as the model calls were associated with a specific model and API version, and even if the underlying off‐the‐shelf models undergo updates over their lifecycle, the configuration used can be recovered. We have no reason to doubt that ever‐increasing model size, and improvements in their training and reasoning processes will improve the reliability of the future models across the classification tasks in line with the scaling laws.

### 6.5 Conclusions From Experiment 2

The Interview Excerpt Task revealed significant differences in question formulation between LLMs and human participants. GPT‐4, when provided with an adapted prompt, outperformed both human groups in the use of invitations and avoidance of option‐posing questions; this is not in line with our hypothesis. However, Llama‐2's performance was less consistent and did not always surpass that of human participants. Contrary to our expectations, there were no clear differences between psychologists and naive participants in this task.

We used an LLM to classify our participant's questions. Interestingly, the process of classifying questions using an LLM proved to be more challenging than anticipated. Despite extensive efforts, not all categories could be accurately sorted without additional rule‐based processing in Excel. This highlights the complexity of question classification in investigative interviewing contexts and the current limitations of LLMs in such specialized tasks. Furthermore, we had to exclude the important category of suggestive questions from our analysis, as determining whether a question is suggestive often requires broader contextual information beyond the question itself. The challenges encountered in question classification also point to areas where further refinement of LLM capabilities is needed for specialized applications in forensic contexts.

## 7 Discussion

Our study evaluated the effectiveness of large language models (LLMs) in formulating questions in tasks relevant to child investigative interviews, comparing their performance to trained humans and naive participants across two experiments: a static Interview Excerpt Task and a dynamic Avatar Interviewing Task. In the Avatar Interviewing Task, we compared an optimal human group (professionals including both psychologists and police officers with avatar feedback training experience) with a non‐optimal group (naive participants with no avatar feedback training experience). For the Interview Excerpt Task, we had a professional group (psychologists) without optimal experience or training in investigative interviewing, and a naive group similar to the one in the avatar situation.

In the Interview Excerpt Task, we hypothesized that LLMs and psychologists would outperform naive human interviewers in formulating recommended follow‐up questions, demonstrating higher compliance with investigative interviewing guidelines. This hypothesis was partially supported. GPT‐4 exceeded expectations, outperforming both human groups in use of invitations and avoidance of option‐posing and other low‐quality questions when a prompt that was adapted to LLMs was used. However, Llama‐2 did not perform as well and did not always outperform humans. There were no clear differences between the psychologists and the naive participants as we had anticipated.

For the Avatar Interviewing Task, we hypothesized that LLMs and trained professionals would use more recommended questions, fewer not‐recommended questions, and a higher proportion of recommended questions compared to naive human interviewers. We also expected LLMs and professionals to elicit more correct and fewer incorrect responses from the child avatars. These hypotheses were not fully supported by our findings. LLM (GPT‐4) performance was comparable to naive participants in several metrics, including the number of recommended questions and the amount of correct and incorrect details elicited. However, LLM struggled with maintaining focus on abuse‐related topics and often got sidetracked in conversations showing difficulty in dealing with reluctant avatars and often failed to persist in exploring abuse‐related topics. Professionals (psychologists and police officers) showed improvement after an intervention consisting of feedback on questions they used, particularly in the use of recommended questions and eliciting relevant details. It's crucial to note that the advantage observed for GPT‐4 in the Interview Excerpt Task might not necessarily translate to the Avatar Interviewing Task if there had been a professional human group with optimal training and experience. The difference in the composition and experience levels of the human groups between the two tasks limits our ability to make direct comparisons.

Issues in question formulation performance of all our participants were familiar from literature on child interviewing: Most questions the humans and Llama asked were not open‐ended invitations but rather directive Wh‐questions. This is in line with less open‐ended questions than recommended used when human professionals interview children even when they have had training on child interviewing with most of the information being gathered with directive WH‐ questions (Baugerud et al. 2023). The composition of focused invitations presents a complex challenge for interviewers. The key lies in creating invitations that are clear, concise, yet simple enough for a child to respond to, often requiring the interviewer to extract the essence of a previous statement for further elaboration. This skill was only mastered by GPT. This is not surprising given that invitations are difficult to formulate and human interviewers can struggle with them, especially when trying to gather specific information about a suspected event (Henderson et al. 2020).

At times, the questions formulated by participants, especially the LLMs, were overly complex for children. Over‐reliance on complex responses has been noted in LLM performance also in other settings, like in the context of a therapeutic discussion (Steenstra et al. 2024). In the avatar interviews paraphrasing, used by some participants as part of an invitation, would also often lead to the question being coded as not recommended due to the length of the sentence structure. This was problematic, since in interviews with real children paraphrasing can be beneficial if it is done correctly (Evans et al. 2010).

In our study, both LLMs as well as humans sometimes resorted to closed questions. Yes/no questions, and those providing options for a reply, do not engage the child's ability to recall information and rather rely merely on recognizing the most familiar option. Recognition questions, which are easier to respond to, lead to more guessing and fewer instances of children saying they don't know (Waterman et al. 2001). This means that while with open‐ended questions the child is required to actively retrieve and articulate specific memories, yes/no and option posing instead offer the possibility of simply confirming, denying, or selecting from options presented by the interviewer (Lyon and Henderson 2021). At times, our participants posed questions classified as closed when they probably intended to ask an open‐ended question: Questions formulated using a modal verb or other indirect structure (could you, would you, do you etc.) and intended as directive, often receive ambiguous and non‐elaborative responses from children, making it difficult to discern which aspect of the question they are addressing (Wylie et al. 2021). Challenge arises from children's potential misunderstanding of yes‐no questions, which might include an embedded open‐ended question that children miss and simply reply yes or no (Lyon and Henderson 2021; Szojka and Lyon 2024). It is important to note that we were not able to guide the LLMs to stop asking questions formulated in such an indirect way (could you… do you…) using only prompting, which is not surprising given how different the conversion style of investigative interviews is from what the models see in their training materials. This question formulation style is common in everyday language and considered polite, but studies show that children regularly treat these questions as yes/no questions instead of providing a more elaborate response (Saywitz et al. 2017; Wright and Powell 2007).

LLM performance in interviewing child avatars seemed comparable to untrained humans in many aspects (metrics such as amount of correct and incorrect details, number of recommended questions). The seemingly good performance warrants a more qualitative analysis of the LLM performance to understand more precisely where its shortcomings were: the main problem was that the LLM ‐Avatar interviews mostly did not involve discussions about anything abuse‐related and some of them made little sense due to all of the deviations from abuse related topics. Part of this is because the avatars are quite “reluctant” to disclose abuse and easily deviate from the topic proposed by the interviewer. Tendency to drift off topic unintentionally or when trying to avoid an unpleasant topic, is common also for really small children (Poole and Dickinson 2014). The time limit was set to 10 min per interview and we do not know how a longer interview would have affected the LLMs ability to gather relevant information. The inability to keep the conversation on point probably explains why the LLM did not uncover incorrect details during the interview.

Dealing with reluctance to disclose was difficult to LLMs. Reluctance is present in many real interviews in children of all ages (Nogalska et al. 2023). Factors like abuse type and demographic characteristics might impact the amount and quality of the reluctance (Lev‐Wiesel et al. 2019; Lev‐Wiesel and First 2018). Teaching LLMs persistence without applying undue social pressure or suggestion is a challenge to be resolved. It can be that in the future, LLMs will be able to help identify patterns that work.

The challenges observed in LLM performance may reflect the fundamental differences between their training paradigm and the demands of investigative interviewing. Modern LLMs are trained through instruction tuning and reinforcement learning from human feedback to be helpful assistants that follow user instructions (Ouyang et al. 2022). This responsive orientation, where models are optimized to provide helpful responses to user prompts, represents a fundamentally different conversational stance than the proactive, strategic questioning required in investigative interviewing. Effective investigative interviewing demands not just language generation capabilities, but also the ability to formulate goal‐directed questions, maintain interview focus, and in an appropriately persistent manner pursue relevant topics despite resistance or distraction. These are skills that diverge from the helpful responsiveness that characterizes instruction‐tuned LLMs.

### 7.1 Limitations

Several limitations should be considered when interpreting the results of this study.

First, in the static Interview Excerpt Task, participants categorized as psychologists had general psychology education and professional backgrounds but were not specifically trained in investigative interviewing protocols. Their psychology training should have provided them competency in understanding child development and communication principles, but this general background may not translate directly to the specialized skills required for optimal investigative interviewing question formulation. This limitation suggests that the performance difference between psychology‐trained and naive participants might have been minimal due to the lack of domain‐specific expertise. The performance of human participants may have been constrained by the experiments design, which included a memory component in the task. Participants were provided with guidance on question formulation but were unable to reference these instructions while formulating their questions. This limitation transformed the exercise from a straightforward application of instructions into a dual‐task challenge, involving both recall of guidelines and their practical application. Consequently, the observed performance might not fully reflect participants' true capabilities in formulating appropriate questions, but rather their ability to remember and apply recently learned principles. While this issue was recognized, the data collection platform used made alternative approaches challenging to implement. However, this constraint may reflect realistic conditions faced by less experienced interviewers who must apply recently learned principles during actual interviews under stressful circumstances, where immediate reference to training materials is typically not available. Consequently, while the observed performance might not fully reflect participants' true capabilities in formulating appropriate questions with full access to guidelines, it may provide insight into their ability to retain and apply recently learned principles under pressure, which is relevant to real‐world interviewing scenarios.

Second, only one LLM (GPT‐4) was used for the Avatar Interviewing Task. Given the rapid advancements in LLM technology, newer models such as the latest versions of Claude LLM, which may perform well enough in Japanese, were not included. This restriction limits our ability to generalize the findings across different LLMs and suggests the need for future research to evaluate a broader range of models.

The behavior of the avatars in the dynamic task was notably challenging, with the avatars being highly reluctant to disclose information. The LLMs, aiming perhaps to be sensitive to the child avatars, often struggled to discern what was relevant and what was not, leading to suboptimal questioning. This difficulty implies that the Avatar Interviewing Task may not have fully showcased the LLMs' potential capabilities in question formulation.

Both tasks in our study required only short‐term memory for interview content. This leaves an open question about how LLMs would perform in scenarios that more closely resemble real‐life interviews, where interviewers must remember extensive details about what the child has said, as well as background information about the case, to formulate appropriate follow‐up questions. This is needed to avoid suggestive questioning and to explore all plausible alternative lines of investigation. The ability to manage and recall large amounts of information over extended periods is a significant challenge for human interviewers and would likely pose a challenge for LLMs. Future research should investigate how LLMs can be enhanced to handle these complexities, potentially through improvements in their memory capabilities and integration with case management systems.

Additionally, several other limitations impact the inferences that can be made from these studies. Some aspects of the dynamic nature of real‐life interviews, including the ability to build rapport and manage non‐verbal cues, were not replicated in the Avatar Interviewing Task. LLMs' inability to process and respond to these non‐verbal cues likely influenced their performance. Moreover, the controlled environment of the static task may have artificially enhanced the LLMs' performance by eliminating distractions and complexities present in actual interviews. Therefore, while LLMs performed well in a static setting, their real‐world applicability remains uncertain.

Lastly, the experiment's reliance on prompt engineering to guide LLM behavior is an inherent limitation in using LLMs for such tasks. The need for precise and effective prompt design underscores the dependency on human expertise to optimize LLM performance, which may not always be feasible in practical applications. To address this, professionals in investigative interviewing and forensic psychology researchers will need to collaborate with LLM experts to create tools that can be used both in training investigative interviewers and assisting them during actual interviews. This collaboration could lead to the development of more effective and practical applications of LLMs in the field, ensuring that these tools are tailored to the specific needs and challenges of investigative interviewing.

### 7.2 Future Directions

An interesting research question for future LLM development relates to identifying the most effective details for focused invitations, aiming to maximize the relevance and quantity of information obtained. Utilizing datasets with real or realistically modeled transcripts could enable LLMs to discern patterns for formulating effective invitations across various child demographics and contexts. Currently, LLMs face challenges in proficiently eliciting abuse‐related information through open‐ended questions. If a model can be trained to pose effective, evidence‐based, and context‐sensitive questions, it could become a valuable tool for real‐world interviews once privacy concerns are addressed. Real‐time support for question formulation during interviews is challenging to arrange, even with a partner or a multi‐professional team present. This tool could be particularly beneficial for interviewers who work alone and lack immediate access to colleagues for suggestions on further questioning.

We only used prompt‐engineering in these tests. LLMs may need to be tested using more sophisticated methods than prompt engineering. For example, retrieval augmented generation (RAG) could be useful in guiding the LLMs in asking appropriate questions in child investigative interview contexts (Fan et al. 2024).

While verbal interaction is a crucial component of investigative interviewing, alternative methods such as drawing can also be beneficial for some children, helping to clarify and elaborate on their narratives (Magnusson et al. 2020). Rapid development of multimodal AI models, which can process and integrate multiple forms of input such as text, speech, and visual data, could allow the LLMs to make use of these alternative methods. As we write this article, advancements in these AI models are occurring at a swift pace, potentially offering new tools that combine verbal and non‐verbal communication to better support children during investigative interviews.

Implementing LLMs in sensitive contexts like child investigative interviewing necessitates addressing ethical concerns, particularly regarding data privacy and the potential for AI bias. Ensuring that LLMs are trained on diverse and representative datasets is important for minimizing these risks.

Even if real‐time application during interviews will not be feasible, an LLM‐based tool that operates locally without compromising confidentiality could still be valuable. The strong performance of GPT‐4 in the static Interview Excerpt Task suggests that LLMs could be made into valuable tools for training interviewers or providing post‐hoc analysis of interview transcripts. They could potentially offer consistent, guideline‐adherent question suggestions, which could be particularly useful for novice interviewers or as a quality control measure. LLM based tools could analyze interviews post hoc to provide reliable feedback on question types, serving as an effective training aid. Its effectiveness can be rigorously tested by comparing interviews conducted with and without this feedback, focusing on improvements in question quality and adherence to best practices. Furthermore, integrating such a tool into training programs could provide a scalable and consistent training solution, enhancing the skills of naive interviewers while adhering to strict privacy standards.

Our study provides initial insights into the potential and limitations of using LLMs in child investigative interviewing tasks. While showing promise in controlled environments, LLMs currently face challenges in dynamic, interactive scenarios. This suggests that, in the near term, LLMs may be most effective as supportive tools for training and analysis rather than as directly being used during child interviews. As LLM technology continues to advance, ongoing research will be needed to fully understand and ethically implement these tools in the sensitive domain of child investigative interviewing. Future studies should include comparisons with optimally trained and experienced human professionals across all tasks to provide a more comprehensive understanding of LLMs' potential in this field. This exploratory research provides groundwork by identifying limitations that must be addressed before LLMs could be responsibly considered for application in child investigative interviewing contexts. While immediate application would be premature, our findings suggest that with proper development and safeguards, LLMs may eventually serve valuable supporting roles. The ultimate goal remains to enhance the quality and effectiveness of these interviews, thereby better serving the needs of vulnerable children in investigative contexts.

## Author Contributions

**Liisa Järvilehto:** conceptualization, methodology, validation, investigation, writing – original draft, writing – review and editing. **Yongjie Sun:** methodology, software, validation, formal analysis, investigation, data curation, writing – original draft, writing – review and editing, visualization. **Nami Aiba:** software, resources, data curation. **Shumpei Haginoya:** software, resources. **Hasse Hallström:** software, writing – review and editing. **Julia Korkman:** supervision, project administration, funding acquisition. **Pekka Santtila:** conceptualization, methodology, validation, formal analysis, investigation, resources, writing – original draft, writing – review and editing, visualization, supervision, project administration, funding acquisition.

## Funding

This study received funding from the Sundell Foundation (Grantee: Julia Korkman).

## Ethics Statement

All ethical requirements concerning the human participant data used in Experiment 1 were addressed and had received prior approval in the original studies. Specifically, the Research Ethics Committee of the Faculty of Psychology at Meiji Gakuin University, Japan, approved the study reported by Haginoya et al. (2023); approval number 20210031). In addition, the Board of Research Ethics at the Department of Psychology, Hosei University, Japan, approved the studies reported by Haginoya et al. (2020), Haginoya et al. (2021) before data collection commenced. Experiment 2 was approved by the IRB of New York University Shanghai (2024‐019‐NYUSH‐New Bund).

## Conflicts of Interest

The authors declare no conflicts of interest.

## Supporting information


Supporting Information S1

## Data Availability

The data supporting the findings of this study are openly available on the Open Science Framework (OSF) at https://osf.io/tegbq/?view_only=f0c09a603eef4c06958a2c7f9d1bf802.

## Appendix A Japanese Language Prompt in Experiment 1

# Mission.

You are a question generator for victim interviewing in child interviewing. Whatever you are given, you will generate only one question consisting of one element. Your goal is to get to the bottom of what happened as best you can.

# Rules of the question

Always follow these rules

1. Ask open‐ended questions and use invitations like “to tell me more about x”. Never ask leading questions that assume or suggest something not mentioned by the child or info given to you about the case.
2. Ask short, concise, targeted questions.
3. Ask cross‐check and follow‐up questions to verify and confirm accuracy and consistency.
4. Ask questions about only one detail or element of the child's narrative per output.

# Context

The user may or may not give you context. If it is given, you may reflect it in the series of questions. If not, ask questions with the task of fully understanding everything that happened.

# ミッション

あなたは児童面接における被害者聴取の質問生成器です。与えられたものが何であっても、あなたは1要素からなるただ1つの質問を生成します。あなたの目標は、できる限り何が起こったのか真相に迫ることです。

# 質問のルール

常に以下のルールに従ってください。

1. オープンエンドの質問をし、「××についてもっと教えて」といった誘いかけを使う。子どもが話していないことや、事件についてあなたに与えられた情報以外のことを想定したり示唆したりする誘導的な質問は決してしないこと。
2. 短く、簡潔に、的を絞った質問をする。
3. クロスチェックやフォローアップの質問をして、正確さと一貫性を検証、確認する。
4. 1回のアウトプットにつき、子どもの語りについて1つの詳細または要素のみについて質問する。

# 文脈

ユーザーが文脈を与えることも与えないこともあります。もし与えられたなら、それを一連の質問に反映させることができます。そうでない場合は、起こったことすべてを完全に理解することを任務として質問してください。

## Appendix B Further Analyses Using Full Data in Experiment 1

In the main analyses, we relied on random sampling such that each human participant contributed either their first or fourth interview. This design choice ensured the statistical independence of observations, as multiple interviews from the same individual would otherwise introduce dependency across measures. To provide a more comprehensive view of the data trends and to assess the robustness of our findings, we additionally conducted analyses including all participants' first and fourth interviews. These results were then compared against the performance of the large language model.

Analyses of the number of recommended questions revealed significant group differences, *F* (4, 139) = 7.99, *p* < 0.001, *η* ^2^ = 0.187. Professionals after receiving feedback training (*M* = 21.14, SD = 9.79) asked significantly more recommended questions than all other groups, including professionals before training, naïve participants both before and after training, and the LLM. The LLM (*M* = 8.69, SD = 3.70) performed at the same level as untrained human participants, but clearly below the trained professionals.

For the number of not‐recommended questions, there was also a significant group effect, *F* (4, 139) = 10.82, *p* < 0.001, *η* ^2^ = 0.237. Professionals after training (*M* = 6.29, SD = 4.57) produced significantly fewer not‐recommended questions than all other groups, whereas the LLM (*M* = 17.69, SD = 4.05) produced significantly more not‐recommended questions than any human group, highlighting its reliance on less desirable questioning strategies.

The proportion of recommended questions confirmed these patterns, *F* (4, 139) = 9.76, *p* < 0.001, *η* ^2^ = 0.219. Professionals after training (*M* = 0.75, SD = 0.19) achieved the highest adherence to recommended questioning practices, significantly outperforming all other groups. The LLM (*M* = 0.33, SD = 0.14) performed similarly to naïve participants before training but worse than naïve participants after training, again falling short of optimally trained professionals.

Turning to the quality of information elicited, group differences emerged for the number of relevant details, *F* (4, 139) = 7.85, *p* < 0.001, *η* ^2^ = 0.184. Professionals after training (*M* = 5.57, SD = 2.69) elicited significantly more relevant details than any other group. In contrast, the LLM (*M* = 1.56, SD = 1.55) generated fewer relevant details compared with trained professionals, and did not outperform untrained humans.

Finally, results for the number of wrong details indicated a significant group effect, *F* (4, 139) = 4.44, *p* = 0.002, *η* ^2^ = 0.113. Interestingly, the LLM (*M* = 0.38, SD = 0.62) produced significantly fewer wrong details than professionals before training and naïve participants before training. Its performance did not differ significantly from professionals after training or naïve participants after training, suggesting that although the LLM was limited in producing high‐quality recommended questions and relevant details, it did not increase the rate of erroneous content.

Taken together, these robustness checks largely confirm the conclusions from the primary analyses. Feedback training markedly enhanced human performance across multiple dimensions of question quality and information elicitation. The LLM, while comparable to untrained novices in some respects and even less error‐prone in generating wrong details, consistently underperformed relative to well‐trained professionals.

## Appendix C System Prompt Version 1 in Experiment 2

### Formulating Questions in Child Investigative Interviews: A Quick Guide

Investigative interviews with children require a delicate balance of achieving comprehensive, accurate information while ensuring the child's comfort and cooperation. This document provides a concise overview of how to formulate questions during child investigative interviews, drawing on the NICHD protocol and best practices in the field.

#### Understanding the NICHD Protocol

The NICHD Interview Protocol is a structured approach developed to improve the effectiveness and reliability of interviews with children, particularly in cases of suspected abuse. Its core principles emphasize open‐ended, non‐suggestive questioning that supports the child in providing accurate and detailed accounts of their experiences.

#### Recommended Question Categories and Examples

Open‐ended questions are inquiries formulated in such a way that they cannot be answered with a simple ^“^yes” or ^“^no,” or with a single fact or detail. Instead, they require a more elaborate response, encouraging the person being asked to provide a narrative, share opinions, feelings, or elaborate on a topic. In the context of interviews, open‐ended questions are instrumental for eliciting comprehensive and detailed information, promoting free expression and a deeper exploration of the subject matter under discussion. Here are the main types of open‐ended questions:

#### Broad Invitations

Definition: Broad invitations are open‐ended prompts that encourage the child to provide as much information as possible about an event or experience without directing their attention to specific details. These questions promote narrative responses and are the most effective way to gather comprehensive information from the child.

Examples:

- “Tell me more about that”
- “Tell me everything that happened from the beginning.”
- “Explain more about your day at the park.”
- “Describe what you do when you're at home.”
- “I'm interested in hearing all about your visit with your dad.”

#### Focused Invitations

Definition: Focused invitations are also open‐ended but target a specific part of the child's earlier narrative or a particular aspect of their experience. Although more directed than broad invitations, they still encourage the child to provide detailed accounts without suggesting specific answers or details.

Examples:

- “You mentioned playing a game. Tell me more about that game.”
- “You talked about a visit to your grandma's house. What did you do there?”
- “You said 'after lunch. Tell me more about what happened after lunch?”
- “Let's go back to when you said 'mom was kind.“’ What made her kind that day?”

#### Utilizing Broad and Focused Invitations

The goal in using both broad and focused invitations is to allow the child to lead the narrative, providing information in their own words and at their own pace. These types of questions minimize the risk of inserting the interviewer's assumptions into the child's account and help avoid inadvertently leading the child to specific answers.

When starting the substantive phase of the interview, it's recommended to initiate with broad invitations to give the child the opportunity to disclose information about the event freely. As the interview progresses, focused invitations help the interviewer delve deeper into specific details mentioned by the child, providing clarity and allowing for a more detailed exploration of critical aspects of their narrative.

#### Directive Questions

Directive questions are straightforward inquiries about specific details of an event or situation. They are aimed at eliciting precise information without leading or influencing the child's response.

Examples:

- “Where did you go with mom?”
- “What game did they play with you?”
- “With whom were you at that time?”
- “What do you like to do at home?”
- “When did you see her?”

#### Clarifications

When a child mentions an emotion or opinion, clarification questions help understand their perspective or feelings related to that statement.

Example:

- After the child says ^“^Mom is kind to me,” you might ask, ^“^In what way is mommy kind to you?” or ^“^what does that mean?”

Questions to Avoid

Overly Complex or Multiple‐Part Questions

Asking about more than one detail or topic in a single question can confuse the child.

- Incorrect: ^“^Where were you with your dad, and what were you doing there?”

#### Using Difficult Terminology

Avoid words or concepts that might be unfamiliar to the child.

- Incorrect: ^“^What obscene activity was done against your genitals?”

Closed questions like yes/no or forced choice questions

Questions that limit the child's response options can lead to inaccurate or distorted information. Also try to avoid questions that can be answered only with yes or no response. Not that also questions formulated “Can you tell me…/can you remember… Do you know… will be classified into this category, even if they continue in an open way and the formulation is meant to be polite. This is because children can, and often do, interpret them in as a yes/no question and provide less information.

- Incorrect: ^“^Didn't you go out with daddy?”
- Incorrect: ^“^Did you see him last night or this morning”
- Incorrect: ^“^Can you tell me more about that”

Expressing Interviewer's Opinions or applying social pressure

Do not ask questions that assume a certain response or let the child know what you want them to reply. Maintain neutrality and professional demeanor throughout the interview.

- Incorrect: ^“^I can't do my work if you don't say anything.”
- Incorrect: ^“^That must have hurt, did it not?”
- Incorrect: ^“^Your friend has told as about this already, so you should too”

#### Conclusion

Effective child investigative interviewing involves carefully crafted questions that are clear, simple, and open‐ended. Avoid complex, leading, or suggestive questions to ensure the child's comfort and to obtain reliable, comprehensive information.

## Appendix D System Prompt Version 2 in Experiment 2

### Mission:

You are tasked with generating questions for interviewing child victims in a sensitive and objective manner. Your primary goal is to facilitate a narrative that allows the child to express the truth of their experience without leading, influence, or closed‐ended inquiries.

### Operating Principles:

Generate Open‐ended Questions: Every question you produce must be open‐ended, designed to encourage detailed narratives or explanations. Avoid any formulation that might prompt a simple yes/no or other closed‐ended response.

You can only ask one question at a time: do not formulate any follow‐up questions and focus on one question that starts with the approved list of beginnings

Approved Question Starters: Your questions must begin with one of the following phrases, listed in order of preference. These starters are chosen to promote thoughtfulness and detail in responses:

“Tell me more about…”

“Tell me about…”

“What happened after/before…”

“Explain how…”

“Continue describing…”

“Go on about…”

“Elaborate on…”

“Then what occurred…”

“Keep going with…”

“Carry on with…”

“And then?”

“Go ahead and describe…”

“What…”

“Who…”

“When …”

“Why…”

Constraints:

No Yes/No Questions: Completely refrain from yes/no questions or those that can be answered with a single word or brief phrase.

Prohibitions: Do not pose any questions that could be seen as leading or suggestive of a specific answer.
