# Supplementary material for: Large Language Model (LLM) and Human Performance in Child Investigative Interviewing Question Formulation Tasks
Source: Behav Sci Law. 2025 Dec 8;44(1):142–63. doi: 10.1002/bsl.70029 (PMC12865673; doi:10.1002/bsl.70029)
Supplement: Supplementary file 1 — Supporting Information S1 [file BSL-44-142-s001.docx]

|  |  | Prompt 1 | | | | | | | Prompt 2 | | | | | | |
| --- | --- | --- | --- | --- | --- | --- | --- | --- | --- | --- | --- | --- | --- | --- | --- |
|  |  | Llama | GPT | Naive | Psychologists | X^2^ | df | p | Llama | GPT | Naive | Psychologists | X^2^ | df | p |
| Case 1 | Facilitator | 0_a_ | 0_a_ | 6_a, b_ | 11_b_ | 518.926^b^ | 15 | <.001 | 0_a_ | 0_a_ | 6_a, b_ | 11_b_ | 1190.607^b^ | 15 | <.001 |
|  |  | 0.0% | 0.0% | 0.8% | 1.5% |  |  |  | 0.0% | 0.0% | 0.8% | 1.5% |  |  |  |
|  | Invitation | 6_a_ | 64_b_ | 62_b, c_ | 37_c_ |  |  |  | 180_a_ | 479_b_ | 62_c_ | 37_c_ |  |  |  |
|  |  | 0.8% | 8.5% | 8.3% | 4.9% |  |  |  | 24.0% | 63.9% | 8.3% | 4.9% |  |  |  |
|  | Directive | 115_a_ | 345_b_ | 384_b, c_ | 398_c_ |  |  |  | 482_a_ | 225_b_ | 384_c_ | 398_c_ |  |  |  |
|  |  | 15.3% | 46.0% | 51.2% | 53.1% |  |  |  | 64.3% | 30.0% | 51.2% | 53.1% |  |  |  |
|  | Option posing | 551_a_ | 227_b_ | 259_b_ | 263_b_ |  |  |  | 20_a_ | 46_b_ | 259_c_ | 263_c_ |  |  |  |
|  |  | 73.5% | 30.3% | 34.5% | 35.1% |  |  |  | 2.7% | 6.1% | 34.5% | 35.1% |  |  |  |
|  | Multiple choice | 0_a_ | 0_a_ | 1_a_ | 3_a_ |  |  |  | 0_a_ | 0_a_ | 1_a_ | 3_a_ |  |  |  |
|  |  | 0.0% | 0.0% | 0.1% | 0.4% |  |  |  | 0.0% | 0.0% | 0.1% | 0.4% |  |  |  |
|  | Other | 78_a_ | 114_b_ | 38_c_ | 38_c_ |  |  |  | 68_a_ | 0_b_ | 38_c_ | 38_c_ |  |  |  |
|  |  | 10.4% | 15.2% | 5.1% | 5.1% |  |  |  | 9.1% | 0.0% | 5.1% | 5.1% |  |  |  |
| Case 2 | Facilitator | 0_a_ | 0_a_ | 7_b_ | 7_b_ | 636.961^c^ | 15 | <.001 | 0_a_ | 0_a_ | 7_b_ | 7_b_ | 1175.097^c^ | 15 | <.001 |
|  |  | 0.0% | 0.0% | 0.9% | 0.9% |  |  |  | 0.0% | 0.0% | 0.9% | 0.9% |  |  |  |
|  | Invitation | 2_a_ | 72_b_ | 49_b_ | 53_b_ |  |  |  | 111_a_ | 490_b_ | 49_c_ | 53_c_ |  |  |  |
|  |  | 0.3% | 9.6% | 6.5% | 7.1% |  |  |  | 14.8% | 65.3% | 6.5% | 7.1% |  |  |  |
|  | Directive | 134_a_ | 405_b_ | 428_b_ | 499_c_ |  |  |  | 558_a_ | 235_b_ | 428_c_ | 499_d_ |  |  |  |
|  |  | 17.9% | 54.0% | 57.1% | 66.5% |  |  |  | 74.4% | 31.3% | 57.1% | 66.5% |  |  |  |
|  | Option  posing | 555_a_ | 216_b_ | 246_b_ | 166_c_ |  |  |  | 68_a_ | 25_b_ | 246_c_ | 166_d_ |  |  |  |
|  |  | 74.0% | 28.8% | 32.8% | 22.1% |  |  |  | 9.1% | 3.3% | 32.8% | 22.1% |  |  |  |
|  | Multiple  choice | 0_a_ | 0_a_ | 2_a_ | 1_a_ |  |  |  | 0_a_ | 0_a_ | 2_a_ | 1_a_ |  |  |  |
|  |  | 0.0% | 0.0% | 0.3% | 0.1% |  |  |  | 0.0% | 0.0% | 0.3% | 0.1% |  |  |  |
|  | Other | 59_a_ | 57_a_ | 18_b_ | 24_b_ |  |  |  | 13_a_ | 0_b_ | 18_a_ | 24_a_ |  |  |  |
|  |  | 7.9% | 7.6% | 2.4% | 3.2% |  |  |  | 1.7% | 0.0% | 2.4% | 3.2% |  |  |  |
| Case 3 | Facilitator | 0_a_ | 0_a_ | 8_b_ | 2_a, b_ | 454.865^d^ | 15 | <.001 | 0_a_ | 0_a_ | 8_b_ | 2_a, b_ | 1558.268^d^ | 12 | <.001 |
|  |  | 0.0% | 0.0% | 1.1% | 0.3% |  |  |  | 0.0% | 0.0% | 1.1% | 0.3% |  |  |  |
|  | Invitation | 4_a_ | 54_b_ | 19_c_ | 23_c_ |  |  |  | 122_a_ | 524_b_ | 19_c_ | 23_c_ |  |  |  |
|  |  | 0.5% | 7.2% | 2.5% | 3.1% |  |  |  | 16.3% | 69.9% | 2.5% | 3.1% |  |  |  |
|  | Directive | 138_a_ | 334_b_ | 431_c_ | 435_c_ |  |  |  | 561_a_ | 189_b_ | 431_c_ | 435_c_ |  |  |  |
|  |  | 18.4% | 44.5% | 57.5% | 58.0% |  |  |  | 74.8% | 25.2% | 57.5% | 58.0% |  |  |  |
|  | Option  posing | 516_a_ | 293_b_ | 270_b_ | 270_b_ |  |  |  | 44_a_ | 37_a_ | 270_b_ | 270_b_ |  |  |  |
|  |  | 68.8% | 39.1% | 36.0% | 36.0% |  |  |  | 5.9% | 4.9% | 36.0% | 36.0% |  |  |  |
|  | Multiple  choice | 5_a_ | 0_a_ | 0_a_ | 0_a_ |  |  |  |  |  |  |  |  |  |  |
|  |  | 0.7% | 0.0% | 0.0% | 0.0% |  |  |  |  |  |  |  |  |  |  |
|  | Other | 87_a_ | 69_a_ | 22_b_ | 20_b_ |  |  |  | 23_a_ | 0_b_ | 22_a_ | 20_a_ |  |  |  |
|  |  | 11.6% | 9.2% | 2.9% | 2.7% |  |  |  | 3.1% | 0.0% | 2.9% | 2.7% |  |  |  |
| Case 4 | Facilitator | 0_a_ | 0_a_ | 8_b_ | 3_a, b_ | 688.796^c^ | 15 | <.001 | 0_a_ | 0_a_ | 8_b_ | 3_a, b_ | 1775.921^e^ | 15 | <.001 |
|  |  | 0.0% | 0.0% | 1.1% | 0.4% |  |  |  | 0.0% | 0.0% | 1.1% | 0.4% |  |  |  |
|  | Invitation | 2_a_ | 115_b_ | 10_a_ | 48_c_ |  |  |  | 126_a_ | 542_b_ | 10_c_ | 48_d_ |  |  |  |
|  |  | 0.3% | 15.3% | 1.3% | 6.4% |  |  |  | 16.8% | 72.3% | 1.3% | 6.4% |  |  |  |
|  | Directive | 101_a_ | 331_b_ | 391_c_ | 448_d_ |  |  |  | 608_a_ | 204_b_ | 391_c_ | 448_d_ |  |  |  |
|  |  | 13.5% | 44.1% | 52.1% | 59.7% |  |  |  | 81.1% | 27.2% | 52.1% | 59.7% |  |  |  |
|  | Option  posing | 601_a_ | 266_b_ | 331_c_ | 242_b_ |  |  |  | 14_a_ | 3_b_ | 331_c_ | 242_d_ |  |  |  |
|  |  | 80.1% | 35.5% | 44.1% | 32.3% |  |  |  | 1.9% | 0.4% | 44.1% | 32.3% |  |  |  |
|  | Multiple  choice | 2_a_ | 0_a_ | 1_a_ | 0_a_ |  |  |  | 0_a_ | 0_a_ | 1_a_ | 0_a_ |  |  |  |
|  |  | 0.3% | 0.0% | 0.1% | 0.0% |  |  |  | 0.0% | 0.0% | 0.1% | 0.0% |  |  |  |
|  | Other | 44_a_ | 38_a_ | 9_b_ | 9_b_ |  |  |  | 2_a_ | 1_a_ | 9_a_ | 9_a_ |  |  |  |
|  |  | 5.9% | 5.1% | 1.2% | 1.2% |  |  |  | 0.3% | 0.1% | 1.2% | 1.2% |  |  |  |
